# Studying Developmental Variation with Geometric Morphometric Image Analysis (GMIA)

**DOI:** 10.1371/journal.pone.0115076

**Published:** 2014-12-12

**Authors:** Christine Mayer, Brian D. Metscher, Gerd B. Müller, Philipp Mitteroecker

**Affiliations:** Department of Theoretical Biology, Faculty of Life Sciences, University of Vienna, Althanstraße 14, A-1090, Vienna, Austria; University of Antwerp, Belgium

## Abstract

The ways in which embryo development can vary across individuals of a population determine how genetic variation translates into adult phenotypic variation. The study of developmental variation has been hampered by the lack of quantitative methods for the joint analysis of embryo shape and the spatial distribution of cellular activity within the developing embryo geometry. By drawing from the strength of geometric morphometrics and pixel/voxel-based image analysis, we present a new approach for the biometric analysis of two-dimensional and three-dimensional embryonic images. Well-differentiated structures are described in terms of their shape, whereas structures with diffuse boundaries, such as emerging cell condensations or molecular gradients, are described as spatial patterns of intensities. We applied this approach to microscopic images of the tail fins of larval and juvenile rainbow trout. Inter-individual variation of shape and cell density was found highly spatially structured across the tail fin and temporally dynamic throughout the investigated period.

## Introduction

Despite the rapidly growing knowledge of the mechanisms underlying embryological development, little is known about how development varies across the individuals of a population. The variational properties of development determine how genetic and environmental variation translate into phenotypic variation in postnatal and adult individuals [1]–[8]. In turn, the population pool of phenotypic variation is the substrate for natural selection and, hence, a major determinant of organismal evolution [8], [9]. The lack of quantitative studies of developmental variation has impeded the long-expected connection of developmental biology with the formal core of evolutionary theory. In addition, modeling developmental variation is key for understanding the multifactorial etiology of many diseases. Genetic and environmental factors that alter the pattern of developmental variation may increase the probability of individuals to pass a threshold towards pathological development [10], [11].

The study of developmental variation has been hampered by the difficulties of measuring the geometry of developing embryos jointly with the spatial patterns of tissue formation and cellular activity. Yet an integrated understanding of organ formation and evolutionary change requires the coordinated study of gene expression, cellular activity, and organismal geometry [1], [7], [12]. In this paper, we present a novel approach that integrates geometric morphometrics and pixel- or voxel-based image analysis into a combined biometric method, allowing for the joint analysis of embryological shape and spatial patterns of tissue properties. For a demonstration, we apply this approach to a set of two-dimensional microscopic images of the tail fins of rainbow trout, but the approach can equally be applied to other imaging and staining methods as well as to three-dimensional images obtained from embryonic specimens [13], [14].

Geometric morphometrics is the state-of-the-art method for biological shape analysis [15]–[19]. It is based on the representation of homologous point locations, curves, and surfaces by landmarks and semilandmarks (two- or three-dimensional measurement points). Semilandmarks are points on curves or surfaces for which the exact position along the curve or surface cannot be determined using anatomical criteria. They are estimated in the course of the analysis, establishing geometric homology within the sample [20], [21]. The careful – usually manual – setting of the landmarks and semilandmarks, based on criteria of anatomical homology, leads to biologically interpretable estimates of means and variances and allows for an effective visualization of such statistical results as actual shapes or shape deformations [15]. However, this limits the application of geometric morphometrics to structures that are present and clearly visible in all individuals of the studied sample. With this method it is not possible to investigate the emergence or loss of structures, which is characteristic for embryological development. Nor does standard geometric morphometrics permit the assessment of structures with unclear boundaries, such as cell condensations or molecular gradients.

Statistical image analysis based on the gray values or color values of image elements (two-dimensional “pixels” or three-dimensional “voxels”) is frequently used in medical imaging [22]–[24]. The variety of image analysis methods differ, among other aspects, in the way images are registered in order to yield correspondence across the compared pixels or voxels. Usually, the registration is an automatic or non-label based (without manual specification of points or curves), non-affine (non-linear) transformation that minimizes some measure of overall dissimilarity across the images [23], [25]. Shape differences between individuals are often considered as nuisance rather than signal and hence are not explicitly estimated. These kinds of approaches have proven powerful for object classification and computer vision, but the imperfect registration of the boundaries of homologous anatomical structures in different individuals may lead to sample averages and variance patterns that are not biologically meaningful [26]. For example, an average of well-delineated structures tends to have fuzzy boundaries, so that this average may no longer represent an actual anatomical structure. The variance of gray values or color values typically is concentrated at the misaligned edges of structures.

In the new method we term *Geometric Morphometric Image Analysis* (GMIA), we take advantage of the strengths of both approaches. It consists of two steps that represent two complementary ways in which developmental differences typically are described in biology. For structures with sharp boundaries, such as organs, bones, and other well-differentiated tissues, morphological variation is described in terms of variation in the shape and in the relative position, size, and orientation of these structures. Structures with diffuse boundaries, such as emerging cell condensations or molecular gradients, instead are described as spatial patterns of intensities or directions (scalar or vector fields) within the organism or within selected organs.

GMIA thus starts with a careful, manual or semi-automatic representation of homologous, well-defined anatomical point locations, curves, and surfaces by the assignment of a dense set of landmarks and semilandmarks. The positions of the semilandmarks are estimated by the sliding landmark algorithm, which minimizes the “bending energy” of the thin-plate spline interpolation, a measure of local form difference, between the specimens and their sample average [20], [21], [27]. The image registration consists of two steps. First, the landmark configurations are superimposed by Generalized Procrustes Analysis (a least squares-based rigid registration plus scaling; [28]), which standardizes for variation in overall position, scale, and orientation. The coordinates of the registered landmarks and semilandmarks, the so-called Procrustes shape coordinates, parameterize the shape of the digitized structures. Second, the actual images are all registered to the sample average shape of the landmark configurations by another use of the thin-plate spline interpolation [27]. In these registered images, the anatomical structures (point locations, curves, and surfaces represented by landmarks and semilandmarks) thus have exactly the same shape in all specimens, and the spaces in between the landmarks are interpolated as “smoothly” as possible (minimizing the integral of the squared second derivatives; see Methods section for details). In the vicinity of the aligned anatomical structures, the registered pixels are likely to represent homologous tissue locations within the sample. Depending on the actual imaging and staining methods, the gray values or RGB values (the values of red, green, and blue color channels) of these pixels represent the tissue properties of the imaged structures.

This approach yields two complementary sets of data: (1) the Procrustes shape variables, describing variation of well-differentiated anatomical structures, and (2) the texture of the registered images (i.e., the pixel or voxel values), representing variation in the spatial distribution of imaged tissue properties. Statistics and resulting visualizations can be computed separately for shape and texture, and also jointly for both.

This two-step approach resembles the separate parameterization of shape and “shape-free” texture in active appearance models and related techniques, which have found wide application in face recognition and some areas of medical imaging [29], [30]. But while active appearance models are aimed at identifying or classifying objects from images that display variation in viewpoint, lighting, and other conditions, geometric morphometric image analysis is a biometric method for the joint analysis of shape and tissue properties in a biological context.

**Figure 1:**
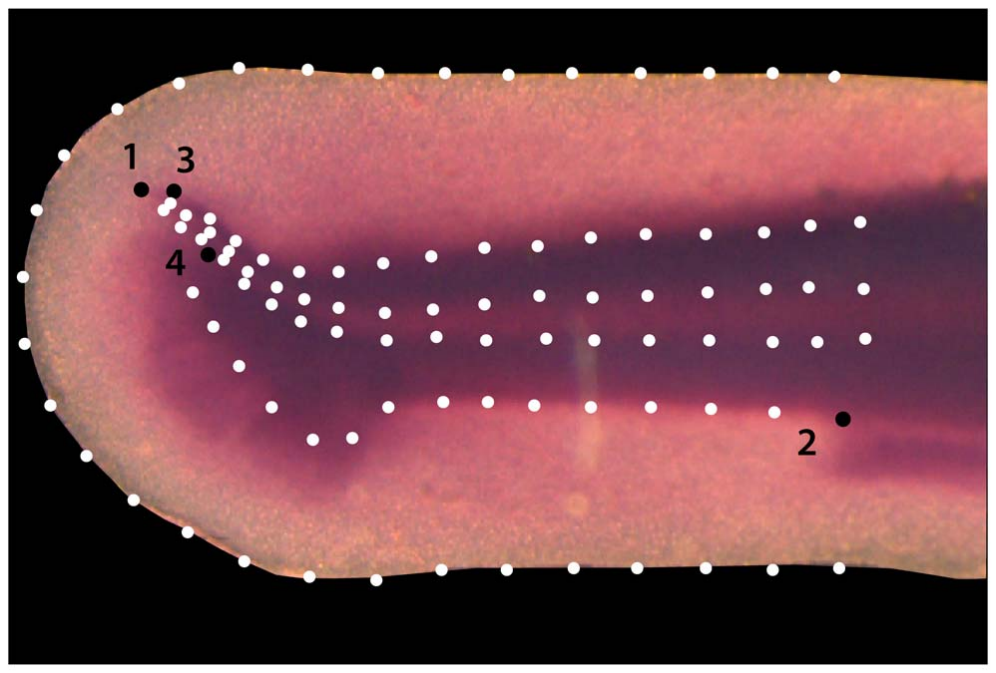
Landmark configuration. Tail fin of a 21 dpf *O. mykiss* specimen with four anatomical landmarks (black points) and 95 semilandmarks (white points). The anatomical landmarks represent the tip of the notochord (1), the posterior end of the anal fin (2), and the two points where the dorsal and ventral outlines of the musculature meet the outline of the notochord (3, 4). The semilandmarks represent the outlines of the notochord, the musculature, and the fin fold. These landmarks were digitized on microscopic images of 20 *O. mykiss* specimens, seven of them fixed at 21 dpf, eight at 40 dpf, and five at 56 dpf.

To illustrate this approach, we use a sample of 20 larval and juvenile specimens of rainbow trout (*Oncorhynchus mykiss*), seven of them fixed at 21 days post fertilization (dpf), eight at 40 dpf, and five at 56 dpf. All specimens were stained in the same way with Mayer's hematoxylin and were stored in 75% glycerol. Microscopic images, prepared under identical conditions, were taken of the tail fins or their corresponding precursors (see Methods section for details). Hematoxylin stains the cell nuclei, hence the color intensity in the microscopic images is taken to correspond with local cell density. We recorded the two-dimensional coordinates of four anatomical landmarks and 95 semilandmarks on each specimen in order to quantify the shape of the developing fin fold, the notochord, and the musculature (Fig. 1).

## Materials and Methods

### Staining and imaging

Our sample consists of 20 rainbow trout specimens (*Oncorhynchus mykiss*) from the zoological collection of the University of Vienna. The fish were collected from one hatchery in the year 2000. Specimens were euthanized by overdose of MS222, fixed in buffered formalin, and stored in 70% ethanol. Seven of them were fixed at 21 days post fertilization (dpf), eight at 40 dpf, and five at 56 dpf. In 2013, the specimens were transferred into distilled water before they were stained with Mayer's hematoxylin for 10 minutes. After keeping them in tap water for 15 minutes, they were transferred to a Scott Solution (tap water, sodium bicarbonate, magnesium sulfate) for 10 minutes, and then again rinsed in tap water for 10 minutes. The stained specimens were stored in 75% glycerol. The tail fins of all specimens were photographed with a Leica MZ16F stereomicroscope. All images were taken under identical light conditions and standardized specimen orientation with a 5× magnification (see S1, S2, S3 Figures). All use of trout specimens are in compliance with EU guidelines for the treatment of vertebrate animals in laboratory research (DIRECTIVE 2010/63/EU on the protection of animals used for scientific purposes). No specific ethical approval was necessary for the use of these collection specimens.

### Landmarks

We recorded 4 anatomical landmarks and 95 semilandmarks on each of the 20 images (Fig. 1) using the software TPSdig (James Rohlf). The anatomical landmarks represent the tip of the notochord, the posterior end of the anal fin, and the two points where the dorsal and ventral outlines of the musculature meet the outline of the notochord. The semilandmarks represent the outlines of the notochord, the musculature, and the fin fold.

The positions of the semilandmarks along their corresponding curves were computed by the sliding landmark algorithm [15], [20], [21], [31], which iteratively “slides” the semilandmarks along tangents to the curve in order to minimize the bending energy of the thin plate spline (TPS) function between each individual and the sample average configuration. The tangent to the curve at each landmark was estimated by a vector connecting the two neighboring landmarks. The TPS function between two sets of landmarks is a non-affine interpolation that maps the landmarks exactly and the in-between space as “smoothly” as possible by minimizing the integral (in all two or three dimensions) of the squared second derivatives, a quantity referred to as bending energy [15], .

In most applications of the sliding landmark algorithm, the curves start and end with anatomical landmarks that constrain the sliding of the semilandmarks. As the curves in our application were all open, we computed the average landmark configuration only once and iteratively slid the semilandmarks against this average. This guaranteed convergence to a non-degenerate mean shape.

### Shape analysis

The 20 configurations of landmarks and semilandmarks were superimposed by Generalized Procrustes Analysis [18], [28]. Thereby the configurations are translated to a common origin (the centroid – the coordinate-wise average of each configuration – is sent to the origin of the coordinate system), scaled to unit centroid size (square root of summed squared distances between every landmark and their centroid), and iteratively rotated to minimize the sum of squared distances among the homologous landmarks. The resulting Procrustes shape coordinates only describe the shape of each landmark configuration because variation in position, scale, and orientation was removed by the Procrustes registration. The Euclidean distance (square root of summed squared differences) between two sets of shape coordinates approximates the Procrustes distance, a measure of shape difference between the corresponding landmark configurations [16], [18].

Group mean shapes are estimated by averages of the shape coordinates. A low-dimensional ordination of shape space was computed by a between-group principal component analysis [32], which is an orthogonal projection of the individual shapes on the principal components of the group means. The between-group PCs thus constitute an orthonormal basis that optimally (in a least-squares sense) represents the Procrustes distances between the group mean. This technique typically leads to a better group separation than ordinary PCA and does not have the problems associated with discriminant analysis [32]. Shape differences between group means are visualized by thin-plate spline deformation grids [15]. These deformation grids are computed by applying the TPS interpolation between the two mean shapes to the vertices of a regular grid that is superimposed onto the template configuration [15], [18], [27].

### Texture analysis

All images were registered to the sample mean shape based on the measured landmarks and semilandmarks by using the thin-plate spline interpolation [27]. In the registered images, the structures digitized by landmarks all have the same shape within the sample, and the space in between the landmarks is interpolated by minimizing the bending energy of the TPS function. The pixels of the registered images hence are assumed to represent corresponding anatomical structures. In order to avoid empty image elements (in areas of expansion) or overlapping pixels (in areas of compression), the images were subject to backward warping (also referred to as unwarping) instead of forward warping. In the typology of image registration techniques devised by Maintz & Viergever [23] this is an intrinsic (landmark-based), non-affine (or curved), local, semi-automatic, monomodal, intersubject registration with directly computed transformation parameters.

Mean cell density was computed as the average of the RGB values of the registered images, computed separately for every color channel of every pixel. For calculating group mean differences and variances of image texture, we transformed the RGB values of each pixel into a scalar value by using the average of the RGB values of each pixel. This average corresponds to the brightness of the pixel and was interpreted as cell density in our application. Group mean differences and variances of image texture (cell density) were visualized by color maps. As for shape, the pattern of individual and group mean differences in texture were ordinated by between-group principal component analysis. Principal components of images are also referred to as eigenimages in the image analysis literature [24], [33] and are least-squares ordinations of the Euclidean distances between the image textures.

### Joint analysis of shape and texture

Group mean shape and mean texture were jointly visualized by unwarping the average texture to the corresponding average shape. Group mean differences for both shape and texture were displayed by superimposing a TPS deformation grid and a color map of texture differences.

A joint ordination analysis of shape and texture is not possible via PCA because the scaling of the pixel values relative to the shape coordinates is ambiguous. While both shape and texture can be separately equipped with a Euclidean metric (but see also [34]), the arbitrary scaling between shape and texture prevents the use of a common Euclidean metric. To approach a common ordination of shape and texture, we instead used a two-block partial least squares analysis (PLS [35], [36]). PLS yields two linear combinations (each with squared coefficients summing up to 1), one for shape and one for texture, that have the maximum possible covariance. When scaling the two linear combinations via major axis regression so that they also correspond in magnitude, the combined scaled coefficient vectors can be interpreted as a common factor of joint variation (for details see [37]). After projecting both shape and texture in the subspaces perpendicular to these vectors, a second pair of vectors can be computed in the same way, and similarly for further dimensions. We used the first two dimensions of such a scaled PLS analysis to represent the joint variation of shape and texture.

All analyses and visualizations of shape and texture were computed in Mathematica 9.0 (Wolfram Research Inc., Champaign, IL, USA)

## Results

**Figure 2:**
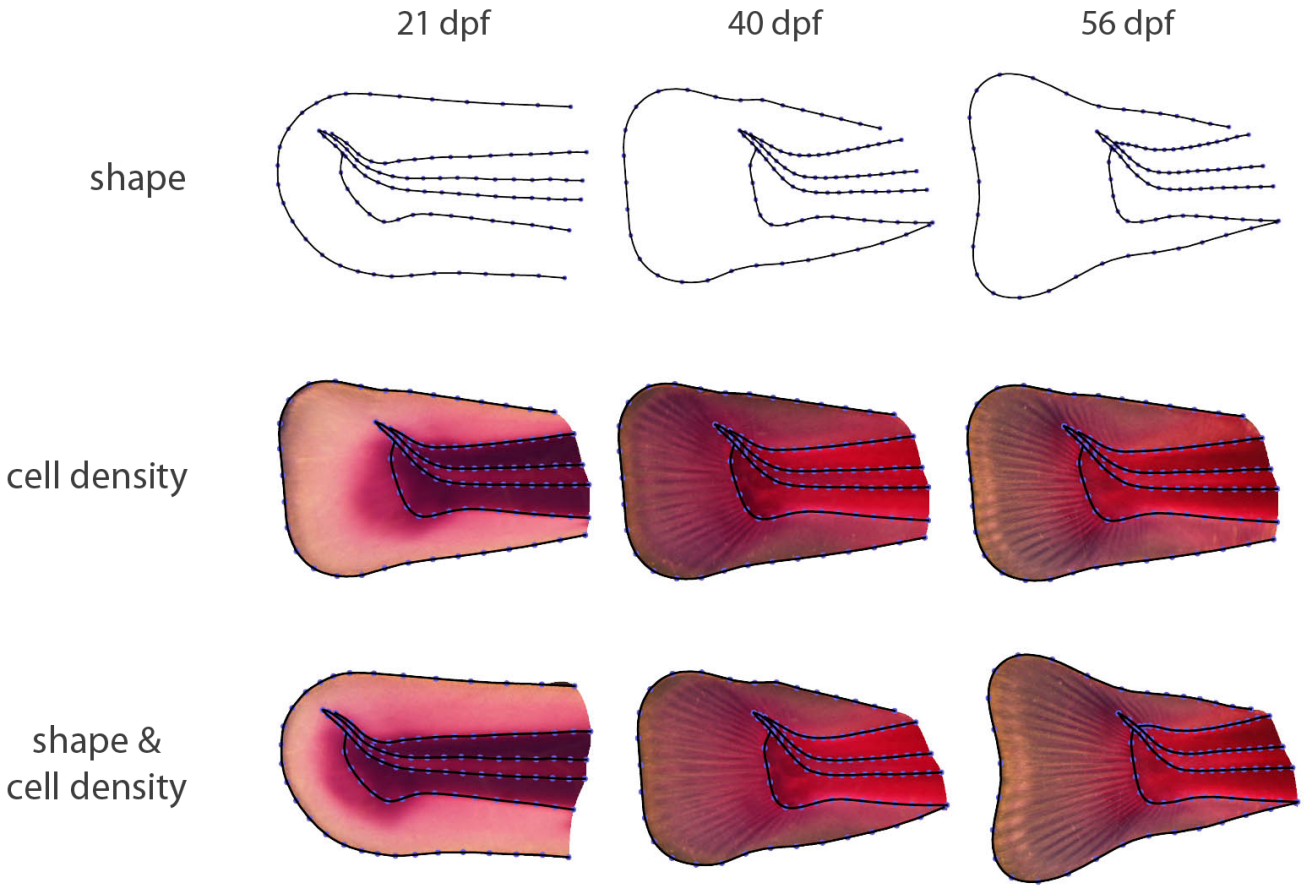
Averages of fin shape, cell density, and both together. Average fin shape (first row), average cell density (second row), and average fin shape together with average cell density (third row) for each of the three age groups (the three columns). The structures measured by (semi)landmarks – the outlines of the fin fold, the musculature, and the notochord – are perfectly registered, but note also how precisely the fin rays, which emerge at 40 dpf, are registered by the TPS interpolation, even though they are not measured by landmarks.

After estimating the semilandmark positions and superimposing the configurations by Generalized Procrustes Analysis, the resulting shape coordinates were averaged for each of the three age groups in order to estimate the *age-specific mean shapes*. Fig. 2a illustrates how the average size of the fin fold and the musculature increased relative to the notochord, and how the fin changed from a rounded to a more triangular shape.

After registering all images to the same shape, texture can likewise be averaged for the three age groups in order to estimate age-specific image texture, which, in our case, represents the *spatial pattern of average cell density*. While no fin rays were visible at 21 dpf, they were partly formed at 40 dpf, and were extended towards anterior at 56 dpf (Fig. 2b). The structures measured by (semi)landmarks – the outlines of the fin fold, the musculature, and the notochord – necessarily are perfectly registered. Note, however, how well the fin rays are registered by the TPS interpolation, even though they were not measured by landmarks (compare these averages with the individual images in S1, S2, S3 Figures).

In Fig. 2c, the average image textures are warped to the corresponding average shape, allowing for the joint visualization of age-specific average shape and average cell density.

**Figure 3:**
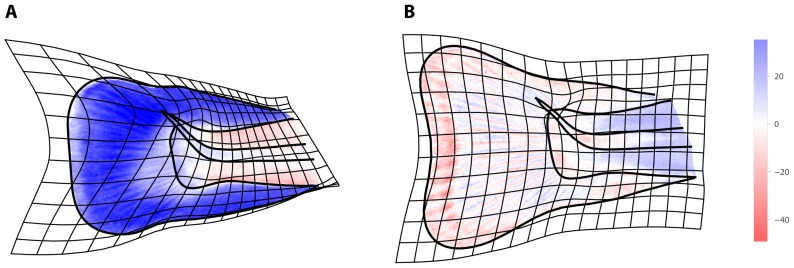
Visualization of average shape change together with average change of cell density. (A) Between 21 dpf and 40 dpf and (B) between 40 dpf and 56 dpf. The deformation grid shows how the shape of the fin changed from a rounded to a more triangular shape during both periods. Changes of cell density are represented by the color map. From 21 to 40 dpf, cell density increased (blue) in the fin, whereas it decreased (red) in the musculature and the notochord. From 40 to 56 dpf, cell density increased in the fin rays and decreased in between.

The differences between the age groups, i.e., the *developmental transformations*, can explicitly be visualized by superimposing a thin-plate spline deformation grid, depicting developmental shape change, and a color map that represents increase or decrease of cell density. For this purpose, the full color information for each pixel was reduced to brightness (average of the RGB values). The higher the cell density (number of projected stained cell nuclei), the darker the pixel will appear and the lower the brightness will be. Fig. 3a shows the transformation from a rounded to a more triangular fin shape between 21 dpf and 40 dpf, which was associated with an average increase of cell density in the fin fold and a decrease of cell density in the musculature and the notochord. Between 40 dpf and 56 dpf, average cell density decreased in the musculature, the notochord, and also in between the fin rays, while density increased inside the fin rays (Fig. 3b).

**Figure 4:**
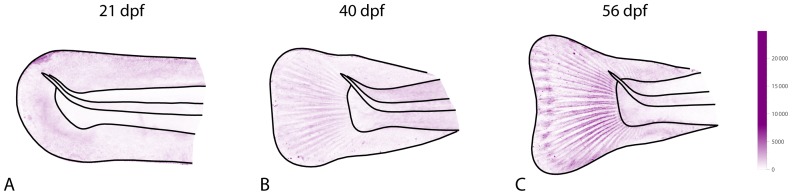
Individual variation in cell density. The spatial pattern of variation in cell density is shown by a color map for each age group. (A) 21 dpf, (B) 40 dpf, (C) 56 dpf. Variation in cell density was not uniform across the tail fin and differed between the age groups. At 21 dpf, the variance was concentrated in the fin fold, whereas cell density was very similar across all individuals in the notochord and in the musculature. At 40 dpf and 56 dpf, variance between the fin rays was higher than variance of cell density in the fin rays.

The spatial pattern of *individual variation of cell density* is also visualized via a color map (Fig. 4). Clearly, variation in cell density was not uniform across the tail fin and differed between the age groups. This reflects different ongoing developmental processes with varying degrees of canalization. At 21 dpf, the variance in cell density was mainly located in the fin fold, whereas cell density in the notochord and in the musculature was relatively similar in all individuals (Fig. 4a). At 40 dpf, variation had increased in the musculature and, after the fin rays had formed, cell density was more variable in between fin rays than within the fin rays (Fig. 4b). At 56 dpf, variance of cell density in the musculature had reduced again, whereas variation in between the fin rays had increased. Note that due to the precise registration, variance is located within the assessed structures, not at their boundaries.

The multivariate pattern of individual differences can be assessed by an ordination analysis that yields a low-dimensional diagram in which the distances between the individuals approximate a certain measure of multivariate (dis)similarity. The shape metric typically used in geometric morphometrics is Procrustes distance (approximated by the Euclidean distance between the sets of shape coordinates [16]). As a metric for image texture, we likewise used the Euclidean distance between the sets of RGB values [33], even though other metrics, e.g. based on mutual information, are equally possible.

**Figure 5:**
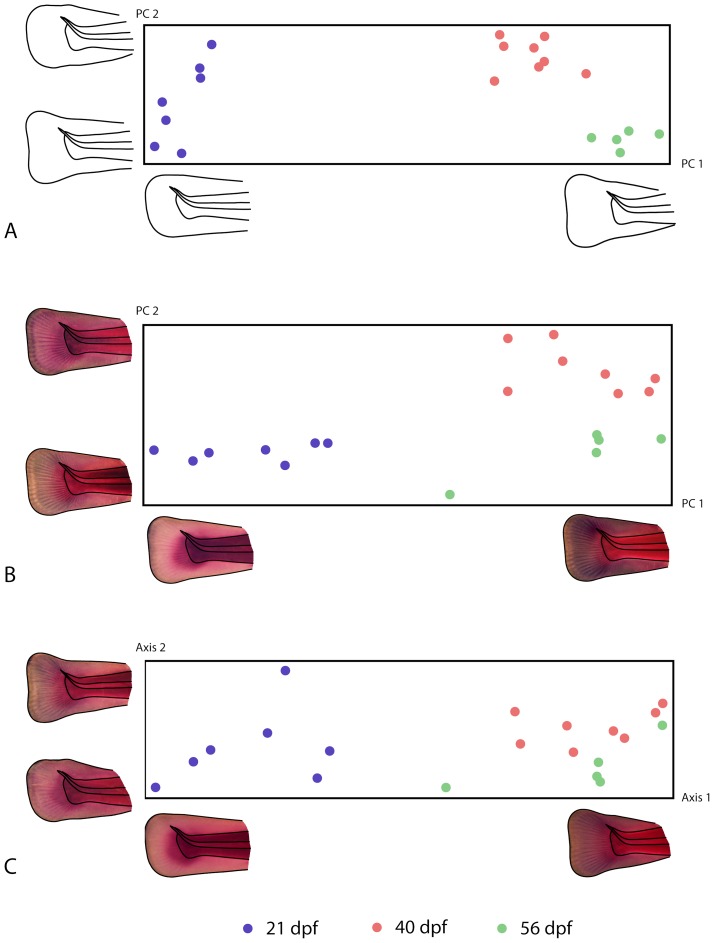
Principal component analyses. Principal component analyses of (A) fin shape and (B) cell density, as well as (C) a joint ordination of both fin shape and cell density based on a scaled partial least squares analysis [37]. Each symbol in the scatter plots corresponds to one individual, and the distance between individuals approximates the overall amount of shape difference or difference in cell density. The axes of these plots correspond to patterns of shape change, to patterns of change in cell density, and to a combination of both, respectively. These patterns are visualized by reconstructed shapes and cell density patterns along the axes that correspond to the limits of variation occurring in the sample (approximately 3 standard deviations from the mean).

We used a between-group principal component analysis [32] to ordinate the multivariate shape differences among the specimens (Fig. 5a). In the scatter plot of the first two between-group principal components (PCs), each symbol represents one individual, and the distance between two symbols approximates the magnitude of overall shape difference between the respective individuals. The first two PCs represent the shape distances among the three group means exactly and account for 91% of total variation between the 20 individual shapes. The shape features corresponding to the two axes are visualized by reconstructed shapes along the corresponding axis locations. The scatter plot shows that – despite considerable individual variation – the three age groups differed in average fin shape and were even separated without individual overlap. The groups differ along PC 1, mainly representing the relative size increase of the fin fold, associated with a shape change from a rounded to a triangular shape. More of these changes had occurred between 21 dpf and 40 dpf as compared to the second period. The second principal component, representing the shape of the fin fold independent of its size, appeared considerably more variable at 21 dpf as compared with the two later stages.

Fig. 5b shows the first two between-group principal components of the registered images, which account for 58% of individual variation in image texture (cell density) and for all the variation between the three group means. In the scatter plot, the symbols represent individuals and the distances between them approximate the magnitude of overall difference in texture. As for shape, the age groups were clearly separated with regard to the spatial pattern of cell density. Within the three age groups, the individuals varied mostly along PC1, which resembles the average differences between 21 dpf and 40 dpf: a shift of cell density from the musculature and the notochord towards the fin fold, along with the formation of fin rays.

Fig. 5c provides a joint ordination of both shape and image texture, based on a scaled partial least squares analysis (see Methods section). The visualizations along the corresponding axes hence comprise differences in fin shape as well as in cell density.

Quantitative developmental studies, such as our example, typically are exploratory; statistical tests of the usual null-hypotheses do not bear much biological relevance here. However, as already obvious from the ordination analyses, permutation tests indicated significant group mean differences in shape as well as in image texture between the age groups (*P*<0.0013 for each of the pairwise comparisons, using the Euclidean distance between group means as test statistic).

## Discussion

Population models in evolutionary theory, genetics, and epidemiology are based on quantitative representations of phenotypic and genetic variation. Yet variation in embryological development and growth – the processes translating genetic variation into phenotypic variation – is still poorly understood. In fact, it has rarely been addressed empirically. Nevertheless, it has been argued on theoretical grounds that the properties of development assume a major role in shaping phenotypic variation within and across populations [1]–[8]. For example, the vast amount of genetic variation is assumed to be “funneled” and structured by the limited number of possible developmental pathways into a much lower-dimensional pattern of phenotypic variation [2], [38]. Strong developmental constraints on the expression of genetic variation may even cause phenotypic stasis [39]. Already in the mid-20th century, C. H. Waddington [40] emphasized the importance of developmental canalization for buffering genetic and environmental variation and the accumulation of cryptic genetic variation, but the actual molecular and developmental mechanisms underlying canalization are still not well understood. The pivotal role of the pattern of developmental variation in humans has recently been adopted by the “Developmental Origin of Health and Disease” paradigm [10], [11].

The study of developmental variation has been hampered by difficulties with the quantification of embryological traits. Our morphometric approach combines the strengths of geometric morphometrics and pixel- or voxel-based image analysis. Well-defined tissue structures are described by the shape of their boundaries, whereas diffuse spatial patterns, such as cell condensations or molecular gradients, are described as scalar fields that are extracted from the texture of the registered images. We demonstrated how to separately analyze embryological shape and image texture (cell density), and we also outlined a strategy for their joint analysis. The anatomical structures are perfectly registered by our method and, hence, group averages of anatomical structures have well-defined boundaries. Variance in image texture is concentrated within the structures, not at their misaligned boundaries [26]. This separate parameterization of shape and image texture resembles the approach in active appearance models and related techniques [29], [30], but the biometric strategy originates from geometric morphometrics [18], [41] and statistical parametric mapping in voxel-based image analysis [22], [24].

In our application of GMIA we analyzed two-dimensional microscopic images. The method can equally be used with other imaging methods, including 3D imaging such as confocal microscopy and micro-CT [13], [14], [42]. Most importantly, it can also be applied to staining methods more specific than the hematoxylin used in our study. Other developmental techniques, such as in situ hybridization or antibody staining, can also be combined with GMIA and will permit the statistical analysis of spatial patterns of gene expression within a population of developing embryos.

Several components of the GMIA approach, most notably the registration of the images in between the landmarks, were based on the TPS interpolation. This algorithm has proved powerful in multiple morphometric contexts, and it worked excellently for registering the fin rays in our application. But the algorithm originated in material physics [27] and may not be optimal in all biological contexts. Likewise, the way in which we transformed RGB values and differences between RGB values into scalar values worked well for our images, but it may need adjustment for other staining and imaging techniques.

By applying GMIA to a sample of rainbow trout tail fins, we were able to demonstrate how average fin shape and average cell density changed within a period of 35 days. These changes include the emergence of the fin rays as novel tissue structures between 21 and 40 dpf, a process associated with a general shift of cell density from the musculature to the fin fold (Figs. 2 and 3). Inter-individual variation of cell density was highly spatially structured and temporally dynamic throughout the investigated period (Fig. 4; compare also [43]). For example, the average increase of cell density within the fin fold between 21 and 40 dpf was accompanied by a reduction (canalization) of variation in cell density in the fin fold and with a temporary increase of variation in the musculature. Between 40 and 56 dpf, variation in the musculature decreased again. The ordination analyses (Fig. 5) demonstrate that the individuals clearly separated between the age groups, even though this separation was more pronounced for shape than for cell density. Within the limits of the small sample, these analyses further indicate that variation along PC2 of shape (i.e., rounded versus triangular shape of the fin fold, independent of its relative size) canalized within the observed age range, whereas no such general trend was apparent for cell density.

In order to verify these results, we produced histological sections along the frontal plane for one fish of each age class. Between 21 and 40 dpf, the tail fin became thicker and the density of mesenchymal cells increased. Also, the number of mucous glands increased. The myotomes decreased in width relative to the notochord. This corresponds well to our results of increasing (projected) cell density in the fin fold and decreasing density in the musculature. Between 40 and 56 dpf, the relative thickness of the myotomes increased again, associated with an increase of visible muscle fibers by approximately 50%. The fin rays and the in-between connective tissue were well differentiated at 40 and 56 dpf. Again, this is represented by our results (Fig. 3).

Highly variable embryonic structures, resulting from variation in the onset, tempo, or mode of developmental processes, are particularly responsive to environmental or genetic disturbances and, hence, are promising candidates for experimental studies. These loci of developmental variation constitute the pattern of phenotypic variation that is subject to natural selection during development and adulthood. But because tissues differ in their degrees of canalization, variation generated at early developmental stages is not equally maintained during later stages and may induce different evolutionary dynamics (see also [44], [45]).

GMIA presents itself as a powerful biometric tool for studying variation in organismal shape in concert with variation in the spatial patterns of various tissue properties. This approach may foster research in an emerging field of biomedical science, the study of developmental variation, which, at the same time, is central to any formal connection between evolutionary and developmental biology. More generally, the inherent strategy underlying GMIA can be used to study the statistical properties of various scalar fields, vector fields, or even tensor fields embedded within in the geometries of different organisms. This includes color patterns, such as in butterfly wings or in human facial skin, or even biomechanical properties, such as the distribution of stress and strain in samples of adult or subadult individuals (e.g., [46], [47]). This capacity of GMIA – the combined registration of variation in embryonic shape, gene expression, and physical properties of cell and tissue masses – can foster the integration of developmental biology and EvoDevo with the population genetic account of evolutionary theory. It may thus provide an important element of an expanded formal framework, or an Extended Synthesis [8] of evolutionary theory.

## Supporting Information

S1 Figure **Images of the 21 dpf specimens.**
(TIF)

S2 Figure **Images of the 40 dpf specimens.**
(TIF)

S3 Figure **Images of the 56 dpf specimens.**
(TIF)
