# Supplementary figures and images for: Studying Developmental Variation with Geometric Morphometric Image Analysis (GMIA)
Source: PLoS One. 2014 Dec 12;9(12):e115076. doi: 10.1371/journal.pone.0115076 (PMC4264869; doi:10.1371/journal.pone.0115076)

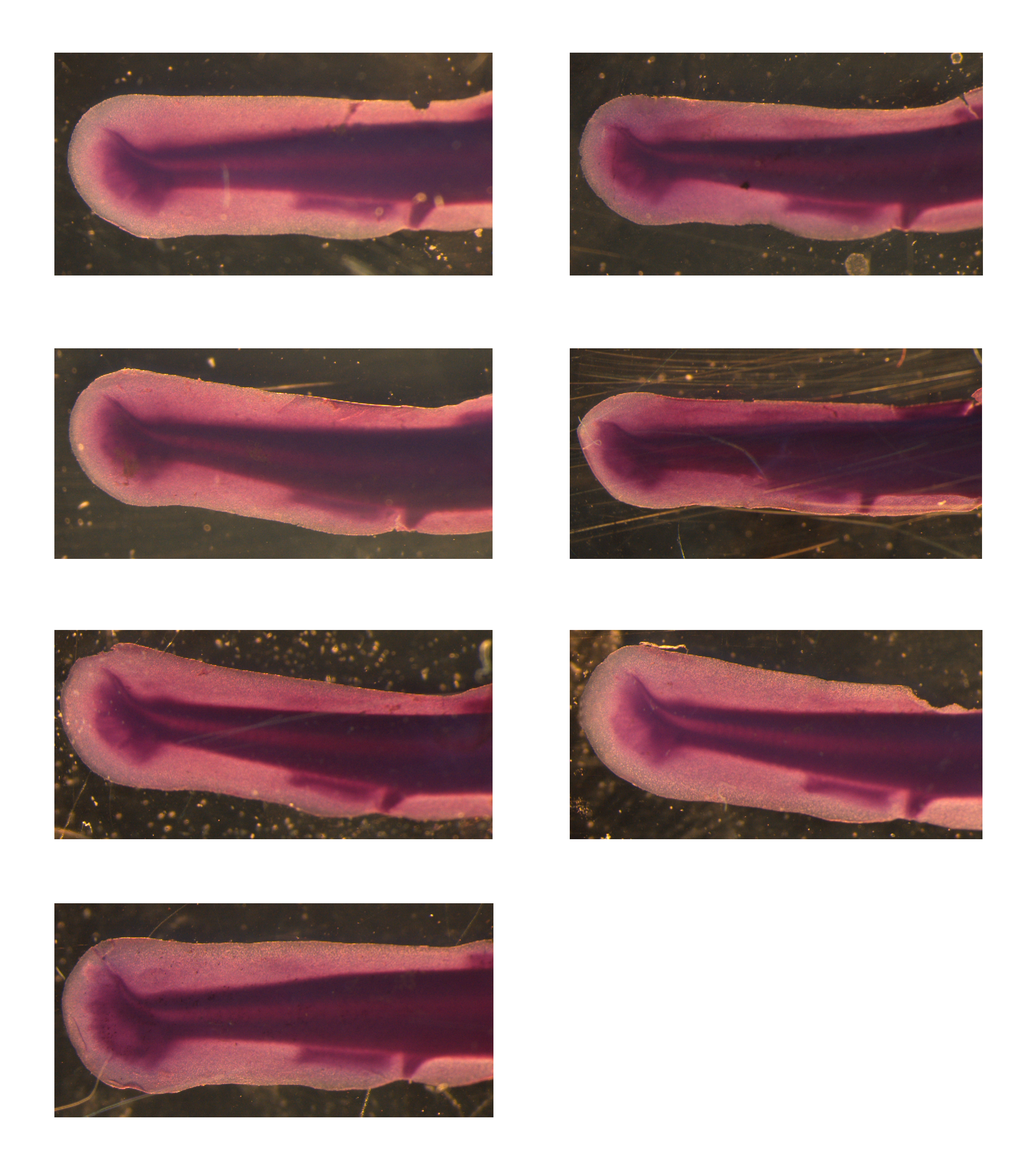

Supplement: S1 Figure — Images of the 21 dpf specimens. (TIF) [file pone.0115076.s001.tif]

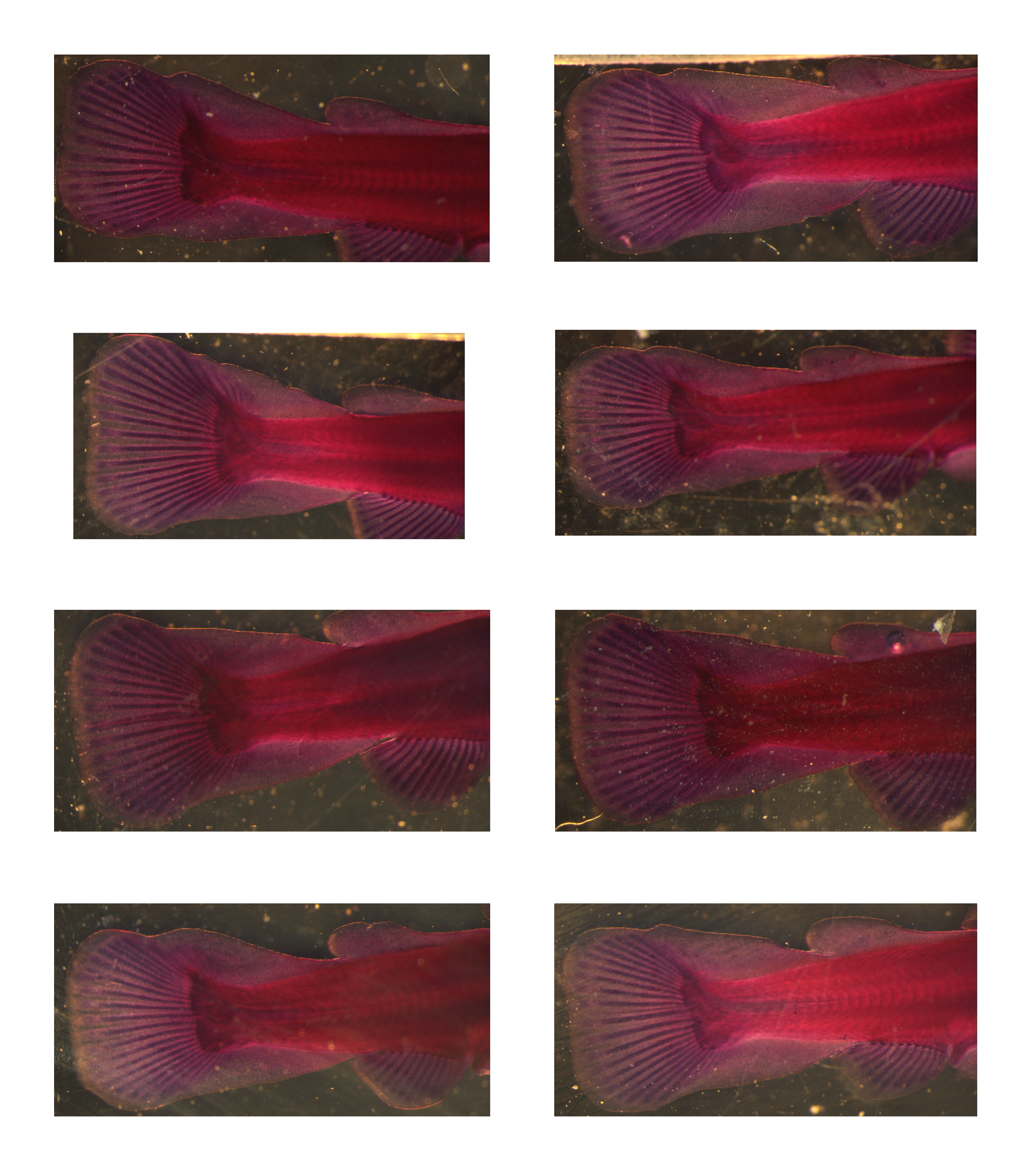

Supplement: S2 Figure — Images of the 40 dpf specimens. (TIF) [file pone.0115076.s002.tif]

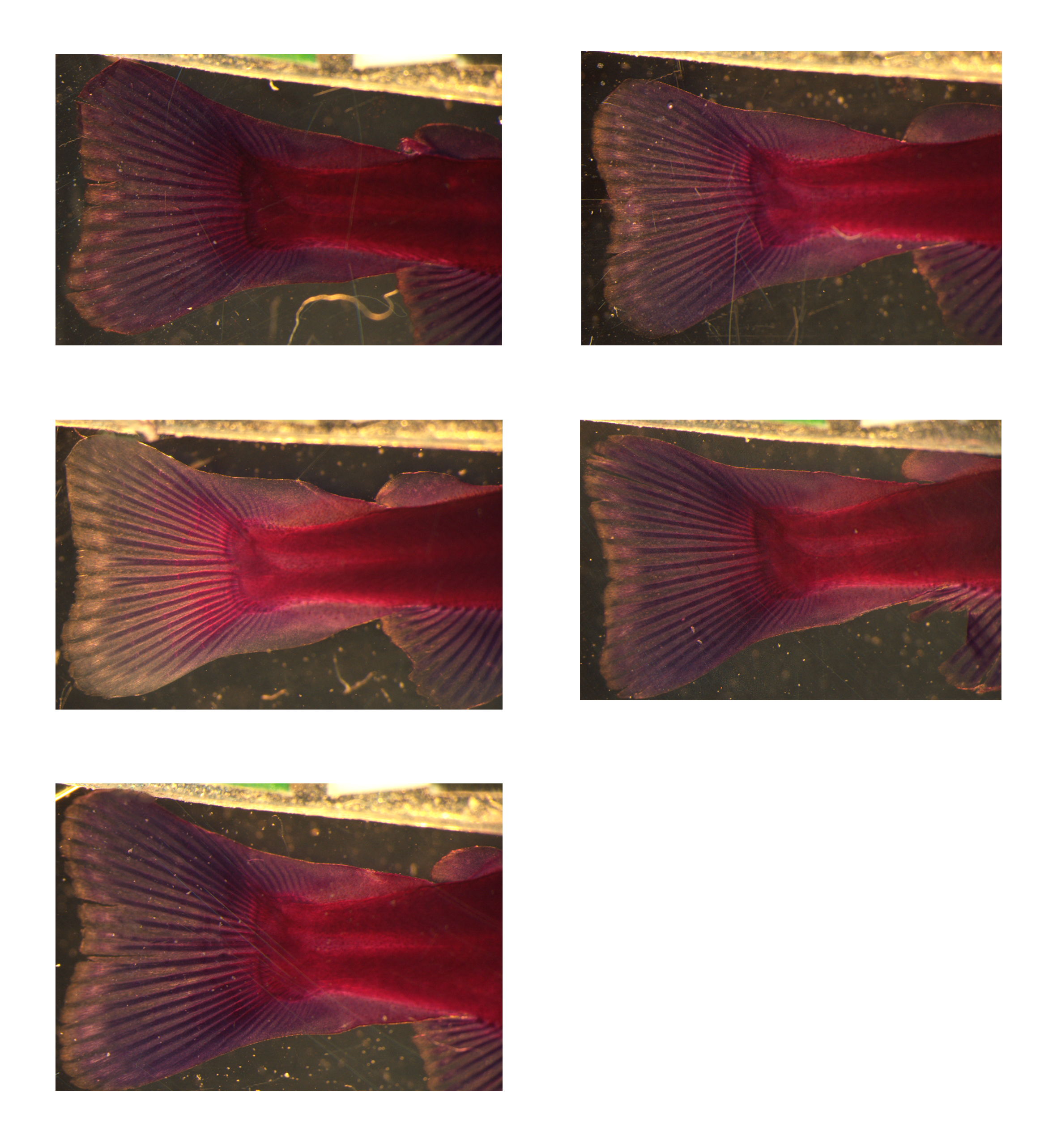

Supplement: S3 Figure — Images of the 56 dpf specimens. (TIF) [file pone.0115076.s003.tif]
